# *Fhb1* disease resistance QTL does not exacerbate wheat grain protein loss at elevated CO_2_

**DOI:** 10.3389/fpls.2022.1034406

**Published:** 2022-11-28

**Authors:** William T. Hay, James A. Anderson, David F. Garvin, Susan P. McCormick, Martha M. Vaughan

**Affiliations:** ^1^ Mycotoxin Prevention and Applied Microbiology Unit, National Center for Agricultural Utilization Research, Agricultural Research Service, USDA, Peoria, IL, United States; ^2^ Department of Agronomy & Plant Genetics, University of Minnesota, St. Paul, MN, United States

**Keywords:** wheat, fusarium head blight, climate resilience, *Fhb1*, elevated CO_2_, grain protein content

## Abstract

Fusarium head blight, a devastating cereal crop disease, can cause significant yield losses and contaminate grain with hazardous fungal toxins. Concerningly, recent evidence indicates that substantial grain protein content loss is likely to occur in wheat that is moderately resistant to head blight when it is grown at elevated CO_2_. Although wheat breeders in North America utilize a number of resistance sources and genes to reduce pathogen damage, the *Fhb1* gene is widely deployed. To determine whether *Fhb1* is associated with the protein content loss at elevated CO_2_, twelve near-isogenic spring wheat lines from either a susceptible or moderately susceptible genetic background, and with, or without the *Fhb1* QTL, were grown at ambient and elevated CO_2_ conditions. The near-isogenic lines were evaluated for differences in physiology, productivity, and grain protein content. Our results showed that the *Fhb1* QTL did not have any significant effect on plant growth, development, yield, or grain protein content at ambient or elevated CO_2._ Therefore, other factors in the moderately susceptible wheat genetic background are likely responsible for the more severe grain protein loss at elevated CO_2_.

## Introduction

Fusarium head blight (FHB), a devastating disease of cereal crops, can cause significant yield losses and contaminate grain with toxins that remain even after typical food processing (Goswami and Kistler, 2004; Bullerman and Bianchini, 2007). In North America, FHB is predominately caused by mycotoxigenic members of the *Fusarium graminearum* (*Fg*) species complex (O’Donnell et al., 2004; Ward et al., 2008). The initial infection begins through the exposed anthers and then the hyphae rapidly infiltrate into the rachis (Brown et al., 2010). There, the pathogen begins producing trichothecene mycotoxins, especially deoxynivalenol (DON), a cytotoxic virulence factor which causes plant cell death ahead of the infection and assists pathogen colonization of the wheat head (Goswami and Kistler, 2004). DON tightly binds to Eukaryotic ribosomes, preventing protein synthesis (Pestka, 2007; Wang et al., 2021). As the infection proceeds, grain yield and quality quickly diminish, leaving withered toxin-contaminated grains unsuitable for food, or feed (Argyris et al., 2003; Awad et al., 2014). Although there are no known wheat varieties that are fully resistant to FHB, a number of gene loci can provide a measure of resistance to the disease (Buerstmayr et al., 2002; Lin et al., 2004; Steiner et al., 2004; Zhu et al., 2019).

Wheat resistance to FHB is a complex quantitative trait controlled by numerous small- to medium-effect quantitative trait loci (QTL) (Buerstmayr et al., 2002; Buerstmayr et al., 2013; Steiner et al., 2019). Despite intensive study, completely FHB-resistant germplasm has not been identified, and only a limited number of QTL have been validated to confer stable FHB resistance (Wang et al., 2020). The most widely used QTL in breeding programs worldwide is *Fhb1*, which originated from Chinese wheat, primarily spring wheat cultivar Sumai 3 (Anderson et al., 2001; Rudd et al., 2001; Buerstmayr et al., 2009; Xue et al., 2011). The *Fhb1* QTL is located on the short arm of the 3B chromosome in wheat populations derived from Sumai 3 (Bai et al., 1999; Waldron et al., 1999; Anderson et al., 2001). In the U.S. and Canada, almost all FHB moderately resistant (MR) hard red spring wheat cultivars currently being used for wheat production have Sumai 3 or its derivatives as an FHB resistance source, and breeding efforts often have focused on selecting genotypes with the *Fhb1* QTL (Hao et al., 2020). While *Fhb1* does not prevent initial *Fg* infection (Type I resistance), it does slow and reduce the spread of the fungal pathogen (Type II resistance) (Lin et al., 2004; Cuthbert et al., 2006; Lin et al., 2006). The identification and functional validation of candidate genes responsible for increased resistance to FHB within the *Fhb1* locus has proven challenging and contentious (Rawat et al., 2016; He et al., 2018; Jia et al., 2018; Soni et al., 2020; Soni et al., 2021). A putative pore-forming toxin-like gene (*PFT)* was identified within the *Fhb1* locus and was predicted to encode a chimeric lectin with two agglutinin domains (Rawat et al., 2016). Transgenic expression of this gene provided a degree of resistance to FHB and the protein encoded by *PFT* was predicted to function as a plant defense protein capable of recognizing fungus-specific carbohydrates and causing membrane damage to potential pathogens. However, in experiments with twelve different wheat varieties of varying levels of FHB resistance the *PFT* gene was found in both FHB resistant and susceptible wheat (He et al., 2018). While the *PFT* gene was associated with, and explains a small part of FHB Type II resistance, it also increased in gene expression in response to abiotic plant stress, methyl jasmonate, abscisic acid, and is likely a part of a multi genic plant defense response.

In Sumai 3, plant defense against FHB is primarily due to the induction of phenylpropanoids, thickening of cell walls that reduce pathogen advancement, and synthesis of antifungal and antioxidant metabolites that reduce pathogen proliferation and DON production (Gunnaiah and Kushalappa, 2014). A recent metabolo-genomics study identified the *TaLAC4* candidate gene in the *Fhb1* locus that is predicted to encode a wheat laccase protein involved in the lignification of secondary cell walls in the wheat rachis (Soni et al., 2020). When the *TaLAC4* gene was silenced total lignin deposition declined, fungal biomass increased, and disease severity worsened. The same research group identified the *TaNAC032* transcription factor involved in regulating lignin biosynthesis, including the *TaLAC4* gene (Soni et al., 2021). When the transcription factor was silenced there was less lignin deposition in the vascular tissues of the wheat rachis and disease susceptibility increased.

Breeders have also introgressed other FHB disease resistance QTLs into wheat, such as *Fhb2, Fhb4, Fhb5, Fhb7* and numerous other minor loci associated with plant defense, kinases, nucleotide-binding and leucine rich repeats (Bent and Mackey, 2007; Brar et al., 2019a; Zhu et al., 2019; Ma et al., 2020). Alone or combined, these loci can contribute to FHB resistance. However, incorporation, and especially stacking of these resistance traits, can have negative pleiotropic effects on yield, grain quality, and grain protein content (McCartney et al., 2007; Brar et al., 2019b). Furthermore, we recently demonstrated a correlation between the degree of wheat FHB resistance and loss of grain nutritional content, particularly grain protein content, at elevated CO_2_ (Hay et al., 2022).

Grain from wheat grown at elevated CO_2_ typically accumulates more carbohydrates and therefore, on a relative basis, contains less protein, minerals, and lipids (Högy and Fangmeier, 2008; Ainsworth and Long, 2021). This alteration in nutritional composition is often referred to as dilution and is caused by enhanced photosynthetic carbon metabolism at elevated CO_2,_ as excess carbohydrates are deposited in the grain as starch (Högy and Fangmeier, 2008; Taub and Wang, 2008; Fernando et al., 2014; Broberg et al., 2017). The loss of grain protein can result in flour that is less nutritious, has reduced baking quality, and compromised end-use utility (Panozzo et al., 2014; Fernando et al., 2015). Beyond impacting food quality, alterations in wheat grain nutritional content at elevated CO_2_ can cause *Fg* to significantly increase mycotoxin biosynthesis, as shown in the MR wheat cultivar Alsen (Hay et al., 2020). Moreover, numerous reports have demonstrated that rising atmospheric CO_2_ is likely to increase wheat susceptibility to FHB (Váry et al., 2015; Vaughan et al., 2016; Bencze et al., 2017; Cuperlovic-Culf et al., 2019). Alarmingly, the deleterious effects of elevated CO_2_ on wheat nutrition were found to be more severe for MR cultivars, compared with susceptible wheat, and was directly correlated with the accumulation of the storage carbohydrate starch (Hay et al., 2022). It was unclear from that study whether *Fhb1* was associated with the decline in grain protein content. While most of impacted cultivars had *Fhb1*, one MR wheat cultivar Bolles, which does not contain *Fhb1*, also had significant protein losses. None of the varieties which exhibited severe protein loss at elevated CO_2_ had *Fhb1* near isogenic lines (NIL) to compare. However, other *Fhb1* NIL wheat lines were readily available for comparison including one set with the Sumai 3 background.

Due to the significant utilization of the *Fhb1* locus for breeding FHB resistance into wheat, it was vital to determine whether *Fhb1* was associated with significant grain protein content loss at elevated CO_2_. Based on our previous results, we hypothesized that another factor in the wheat genetic background, not *Fhb1*, was responsible for the loss in grain protein content. To test this hypothesis, two sets of near-isogenic wheat lines from either a susceptible or moderately susceptible genetic background, and either with (Fhb1+), or without (Fhb1-), the *Fhb1* QTL (**Table 1**), were grown in a completely random block design at ambient (400 ppm) or elevated (1000 ppm) CO_2_ conditions. In addition to grain protein content, the near-isogenic lines were evaluated for differences in development, growth, and productivity. Differences between wheat genetic background or the presence of *Fhb1* were used to evaluate whether either was associated with loss of grain protein at elevated CO_2_.

**Table 1:** Breeding pedigrees for wheat genotypes in the current study.

Genotype	Background	*Fhb1* QTL	Pedigree
260-4	MSB	–	Sumai 3/Stoa RIL 63–4//MN97448
HR 45	MSB	–	Sumai 3/Stoa RIL 63–4//MN97448
HR 123	MSB	–	Sumai 3/Stoa RIL 63–4//MN97448
260-2	MSB	**+**	Sumai 3/Stoa RIL 63–4//MN97448
HR 56	MSB	**+**	Sumai 3/Stoa RIL 63–4//MN97448
HR 58	MSB	**+**	Sumai 3/Stoa RIL 63–4//MN97448
Apogee	SB	–	Apogee
Norm	SB	–	Norm
Wheaton	SB	–	Wheaton
A73	SB	**+**	Apogee*5/Sumai 3: BC_4_F_3_
N1	SB	**+**	Norm*5/Sumai 3: BC_4_F_3_
W4	SB	**+**	Wheaton*5/Sumai 3: BC_4_F_3_

Genetic background of wheat genotypes, as defined by whether the parental cultivars are moderately susceptible (MSB) or susceptible (SB) to FHB infection, and whether a genotype has (+), or does not have (-) the *Fhb1* QTL.

## Materials and methods

### *Fhb1* near-isogenic lines

This study employed two sets of NILs to evaluate the effects of *Fhb1* in FHB susceptible or moderately susceptible wheat genetic backgrounds. The first set of NILs include the hard red spring wheat cultivars Norm, Wheaton, and Apogee. Norm (Busch et al., 1993) and Wheaton (Busch et al., 1984) were developed by USDA-ARS and the Minnesota Agricultural Experiment Station, and Apogee (Bugbee et al., 1997) was developed at Utah State University. Norm and Wheaton long have served as susceptible checks in FHB research, while Apogee has been proposed as a model for FHB research because of its short stature, rapid life cycle, and high level of FHB susceptibility (Mackintosh et al., 2006). Near-isogenic lines harboring *Fhb1* developed for each of these cultivars were also employed in this study. These were generated first by crossing Sumai 3 as the donor of *Fhb1* to each cultivar. A simple sequence repeat molecular marker locus linked to *Fhb1*, *Xgwm493* (Röder et al., 1998), was then employed to select for the presence of *Fhb1* over the course of four generations of marker-assisted backcrossing, with the cultivars serving as recurrent parents. In each cultivar’s backcross pedigree, BC_4_F_1_ plants were surveyed for heterozygosity at *Fhb1*, based on the genotype of the linked molecular marker. A heterozygote within each cultivar’s backcross pedigree that had morphological similarity to the recurrent parents was self-pollinated, and from each resultant BC_4_F_2_ family a single plant homozygous for *Fhb1* was identified and grown to maturity to obtain a BC_4_F_3_ *Fhb1* near-isogenic line for each cultivar. These *Fhb1* near-isogenic lines are designated N1 (Norm near-isogenic line), W4 (Wheaton near-isogenic line, and A73 (Apogee near-isogenic line). These NILs are predicted to be more than 95% genetically identical to their respective parental cultivars; each backcross (BC) generation increases recurrent parent homozygosity by 50% of the remaining heterozygous loci. Self-fertilization, or selfing, increases recurrent parent homozygosity by 25% of the existing heterozygous loci. For example, by BC_4_, the NIL with *Fhb1* would be approximately 94% homozygous for the recurrent parent genome. Selfing a BC_4_F_1_ plant would increase this to 95.5% or so in a BC_4_F_2_ progeny and selfing a BC_4_F_2_ plant would increase this to more than 97%. For this manuscript, this set of NILs is defined as from a susceptible genetic background (SB), due to each parental cultivars’ salient susceptibility to FHB infection. In previous experiments, Norm and Wheaton did not have inordinate grain protein loss at elevated CO_2_, as compared with the significant protein decline observed in some wheat cultivars more resistant to FHB (Hay et al., 2020; Hay et al., 2022).

The second set of NILs was developed during the fine mapping of *Fhb1* (Liu et al., 2006). These six lines, designated as 260-2, 260-4, HR 45, HR 56, HR 58, and HR 123 all have the pedigree (Sumai 3/Stoa RIL 63–4//MN97448) and were derived from a single F_7_ plant that was heterozygous for *Fhb1*. This NIL set possesses some degree of FHB resistance but were developed to have a genetic background which was only moderately susceptible to FHB; moderate susceptibility to FHB was necessary to characterize the effect of *Fhb1* on disease resistance for mapping the genomic region harboring *Fhb1*. For this manuscript, the set of NILs from the Sumai 3/Stoa RIL 63–4//MN97448 pedigree are defined as from a moderately susceptible genetic background (MSB) for comparison with the set of SB NILs derived from Norm, Wheaton and Apogee.

### Growing conditions and evaluating productivity

To evaluate how the presence, or absence, of the *Fhb1* QTL impacted wheat grain protein content, the various wheat genotypes (**Table 1**) were grown in PGR15 environmentally controlled growth chambers (Controlled Environments INC., Manitoba, Canada). The wheat genotypes were grown in a completely random block design, with the growth chambers blocked into four pairs, each block containing a chamber set to ambient [CO_2_] (420 ± 20 ppm, a[CO_2_]) and a chamber set to 1000 ± 20 ppm [CO_2_] (e[CO_2_]). For each genotype, eight seeds were sown in a 20 × 15-cm plastic pot, filled with approximately 4 L of SunGrow Horticulture potting mix (Agawam, MA, U.S.A.), and thinned to 5 plants shortly after seedling emergence. Growth chambers were programmed to a day/night cycle of 25/23°C, respectively, with a 14 h photoperiod at 550 μmol m^−2^ s^−1^ photosynthetic photon flux density from incandescent and fluorescent light sources. The relative humidity was maintained in the range of 50-60% throughout the experiment. The plants were watered daily, and plant positions were randomized after each watering. Additionally, plants received a biweekly fertilization with soluble Peters 20-20-20 nutrient supplement (The Scotts Company, Marysville, OH, U.S.A.) until flowering. The developmental timings of heading (Feekes 10.2), flowering (Feekes 10.5.2), and maturity (Feekes 11.3) were recorded. Seed filling days were determined as the number of days from flowering to maturity. Tiller height and total number of tillers were evaluated after physiological maturity (Feekes 11.3), and grain was harvested for yield after ripening (Feekes 11.4). Remaining wheat straw was collected to gravimetrically determine above ground biomass. Wheat grain moisture and protein content was assessed by a DA 7250 near-infrared (NIR) analyzer (Perten Instruments, Springfield, IL). All local and national regulations were followed, and all relevant permissions were acquired for wheat cultivation and harvest; no genetically modified plants were used.

### Statistical analyses

Results were evaluated by a generalized linear mixed model analysis of variance, with paired growth chamber blocks as a random effect (JMP V15.0), to determine significant differences between genotypes and wheat genetic background due to the effects of elevated CO_2_ (α = 0.05). Details on pairwise comparisons can be found within the table and figure legends. Principal component analysis was performed in JMP V15.0. Additionally, a permutational multivariate analysis of variance was performed in R 4.2.1 (“Prairie Trillium” release, ‘Vegan’ R package 2.6-2) to determine how variation was attributed to the experimental treatments.

## Results

### Effects of elevated CO_2_ on plant development, yield, and grain protein

**Figure 1:**
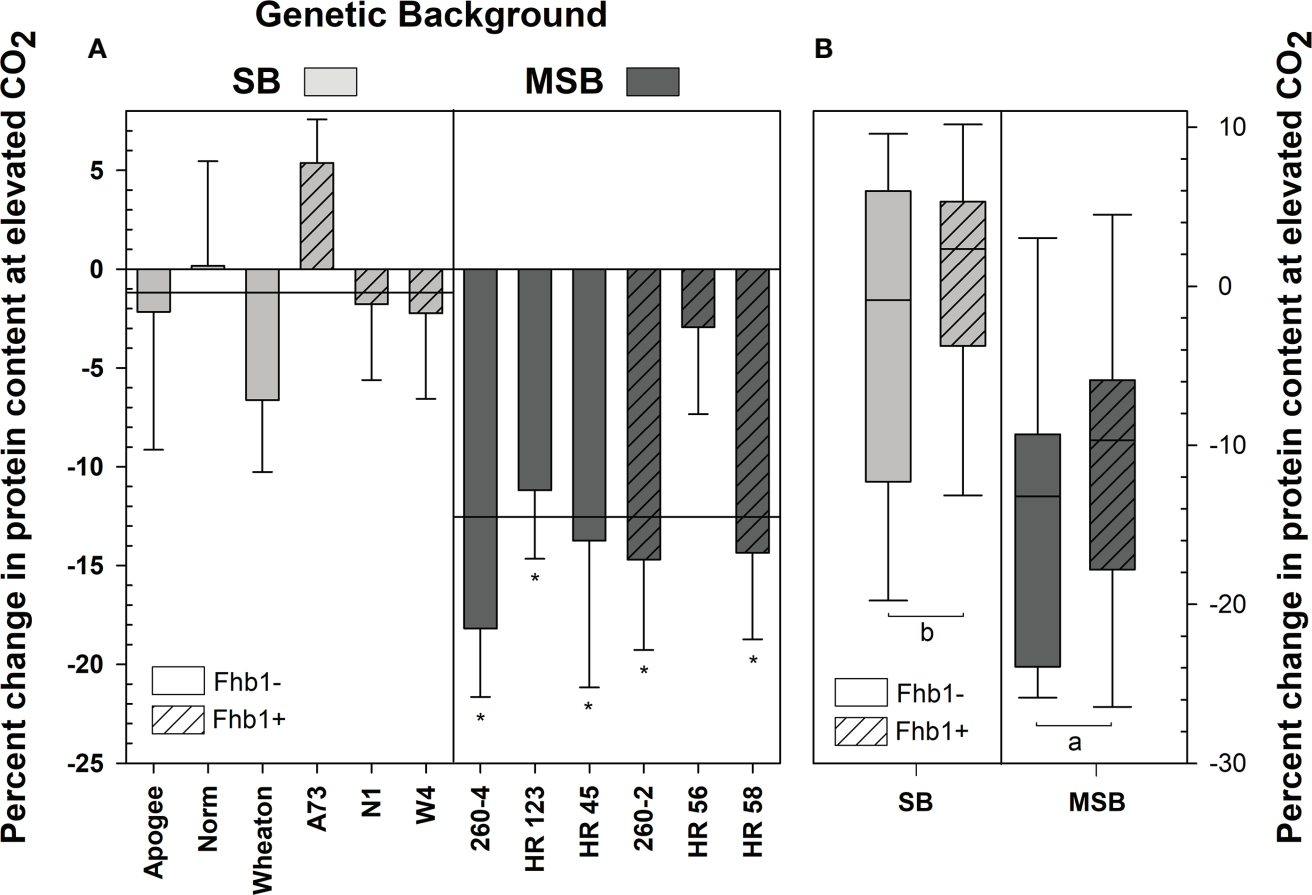
Percent change in grain protein of wheat from a *Fusarium* head blight (FHB) moderately susceptible (MSB) or susceptible (SB) genetic background with (Fhb1+) or without (Fhb1-) the *Fhb1* QTL, grown at elevated CO_2_ (e[CO_2_]) or ambient (a[CO_2_]). **(A)** Percent change in grain protein content of wheat genotypes at e[CO_2_]. Horizonal lines represent the average percent change in grain protein at e[CO_2_] for each genetic background, error bars represent standard error. Asterisks (*) denote statistically significant differences in grain protein content at e[CO_2_] versus a[CO_2_] for a respective genotype as determined by a Student’s t Test (P<0.05; *n* = 4), performed after a significant genotype × [CO_2_] interaction was found. **(B)** Percent change in protein content by genetic background and the presence of *Fhb1*. Different letters denote statistically significant differences as determined by an ANOVA (P<0.05); *n* = 24 (JMP V15.0).

Grain protein content was strongly affected by elevated CO_2_, particularly in MSB wheat, with significant interactions in both genotype × [CO_2_] (P = 0.0038) and wheat genetic background × [CO_2_] (P <0.0001). The *Fhb1* QTL was not a significant contributing factor to differences in grain protein content (P=0.2112). In ambient conditions, wheat grain contained equivalent protein content (P = 0.1351), with the MSB and SB wheat having 16.95% and 16.21% grain protein content, respectively. At elevated CO_2_, every MSB wheat genotype, except HR 56, had significant losses of grain protein content (-12.5% on average), whereas the grain protein content of SB was not impacted (-1.2% on average; **Figure 1A**), when compared with respective genotype at ambient conditions. Grain protein loss at elevated CO_2_ was consistently worse in the MSB genetic background (P = 0.0002; **Figure 1B**), with *Fhb1* having no impact, as no significant genetic background × [CO_2_] × *Fhb1* three-way interaction was found (P = 0.8562).

**Figure 2:**
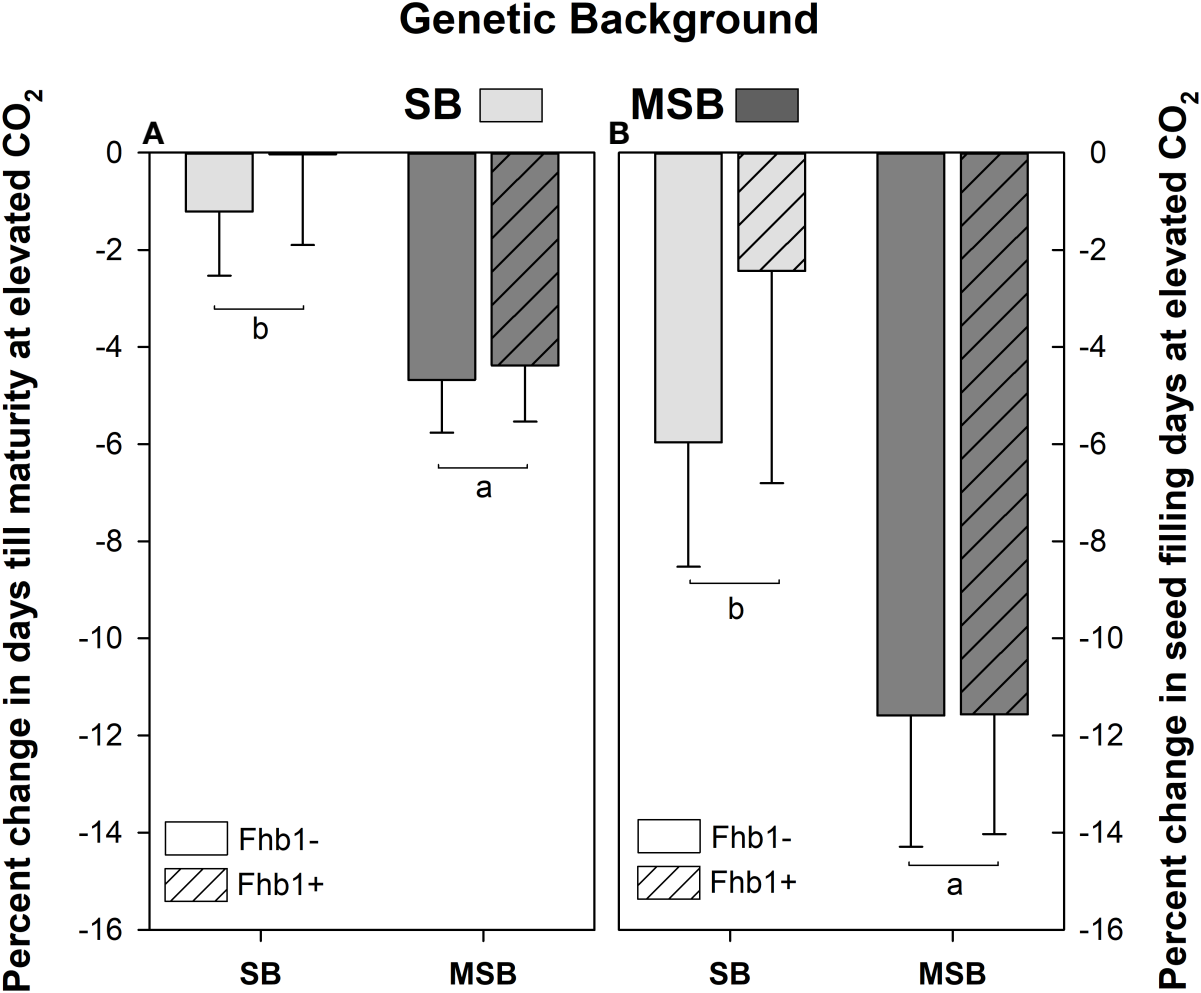
Percent change in seed filling days and days till physiological maturity for *Fusarium* head blight (FHB) moderately susceptible background (MSB) or susceptible background (SB), with (Fhb1+) or without (Fhb1-) the *Fhb1* QTL, grown at elevated CO_2_ (e[CO_2_]) versus ambient (a[CO_2_]). **(A)** Percent change in days till physiological maturity at e[CO_2_], **(B)** Percent change in seed filling days at e[CO_2_]. Error bars represent standard error. Different letters denote statistically significant differences as determined by an ANOVA (P<0.05); *n* = 24 (JMP V15.0).

While developmental timings varied greatly by genotype, wheat heading and flowering were not significantly impacted by *Fhb1* or plant growth at elevated CO_2_. However, the number of seed filling days, and the number of days till physiological maturity (Feekes 11.3) were significantly reduced at elevated CO_2_ for the MSB wheat, compared with the SB (**Figure 2**).

**Figure 3:**
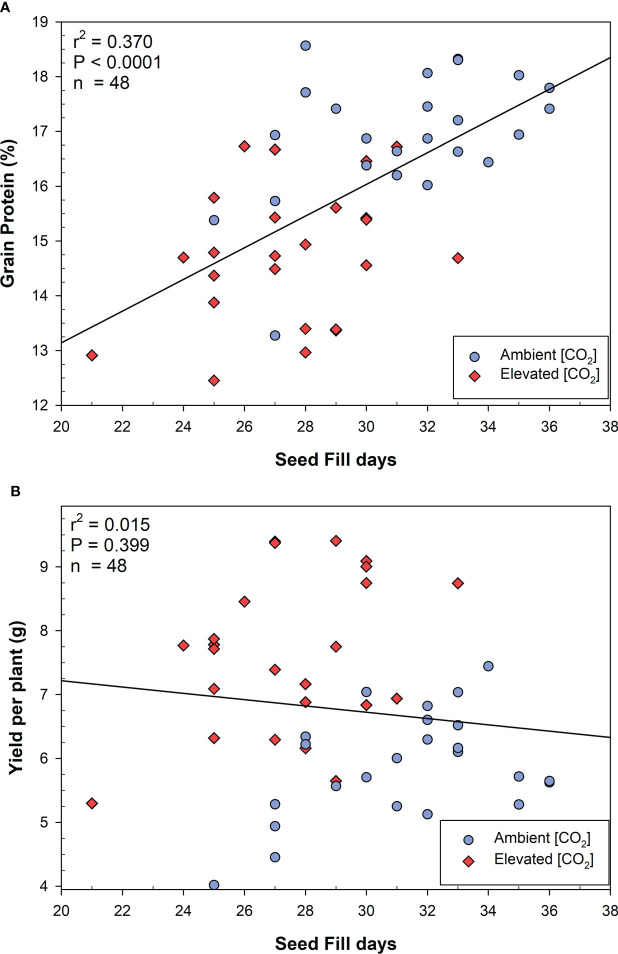
Linear correlations between **(A)** seed fill days and grain protein content, **(B)** seed fill days and yield per plant in moderately susceptible wheat. Linear fits were produced, and the analysis of variance was performed using JMP V15.0.

For the MSB wheat, the number of seed filling days was correlated with grain protein content across all CO_2_ conditions (**Figure 3A**), however growth at elevated CO_2_ significantly reduced both grain protein and seed filling days. The total seed filling days were not significantly correlated with yield in MSB (**Figure 3B**). MSB wheat yields increased at elevated CO_2_ even as the total seed filling days and protein content declined. When examining MSB wheat only at e[CO_2_] (**Supplementary Figure 1**), the number of seed filling days were not significantly correlated with grain protein content (r^2^ = 0.093; P=0.146). Therefore, the reduced number of seed filling days at elevated CO_2_ is associated, but not necessarily the direct cause of reduced grain protein content in MSB.

**Figure 4:**
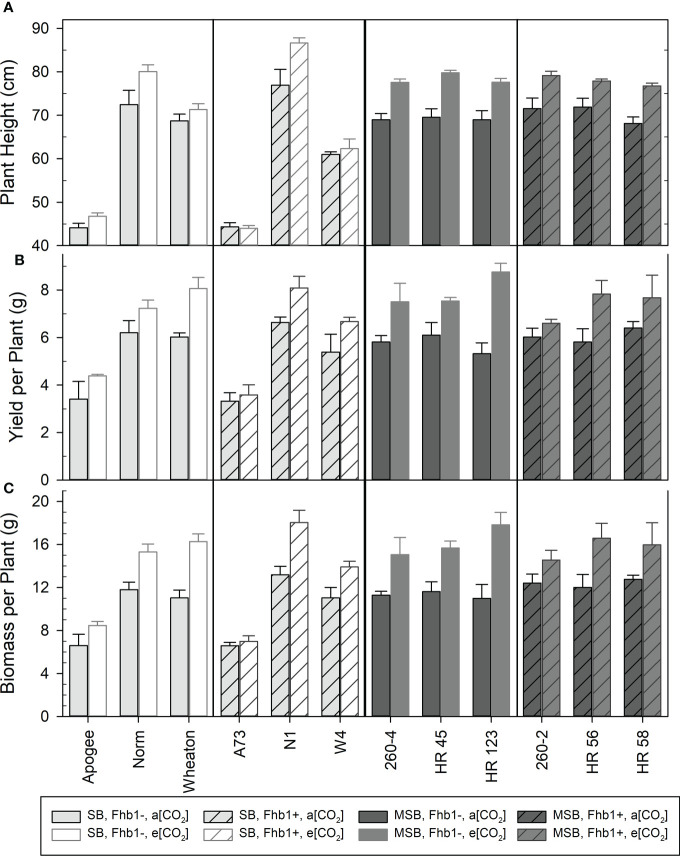
Mean plant height **(A)**, yield per plant **(B)**, and above ground biomass per plant **(C)** for various genotypes, with (Fhb1+), or without (Fhb1-), the *Fhb1* QTL, from either a moderately susceptible (MSB) or susceptible (SB) wheat genetic background at ambient (a[CO_2_]), or elevated (e[CO_2_]) carbon dioxide concentration. Error bars represent standard error.

Wheat grown at elevated CO_2_ had significantly increased plant height (P <0.0001), above ground biomass accumulation (P <0.0001), and yield per plant (P <0.0001). Although MSB wheat grain protein content (% protein) severely declined at elevated CO_2_ (**Figure 1**), the improved yield caused the total amount of harvestable grain protein per plant to significantly increase at elevated CO_2_. At a[CO_2_] wheat had an average of 0.949 g protein/plant of total grain protein, but at e[CO_2_] this average increased to 1.065 g protein/plant (P = 0.0019). While there were significant genotype differences, particularly due to the superdwarf habit and rapid life cycle of Apogee and A73, *Fhb1* had no significant impact on these physiological characteristics and there were no significant genetic background × [CO_2_] interactions (**Figure 4**). The average seed weight was not impacted by growth at elevated CO_2_, and therefore differences in yield were not due to changes in seed weight. Yield increases were most likely due to an increase in the number of tillers per plant, with a 27% increase at elevated CO_2_ for all genotypes (P<0.0001), an average increase of approximately one additional tiller per plant. There was no significant impact of *Fhb1* on tiller number (P = 0.787), nor a significant *Fhb1* × [CO_2_] interaction (P = 0.630). Furthermore, there was no significant genetic background × [CO_2_] interaction (P = 0.422), and therefore, the increase in tiller number at elevated CO_2_ was not associated with protein loss in MSB.

### Impact of *Fhb1* or genetic background on wheat characteristics

**Figure 5:**
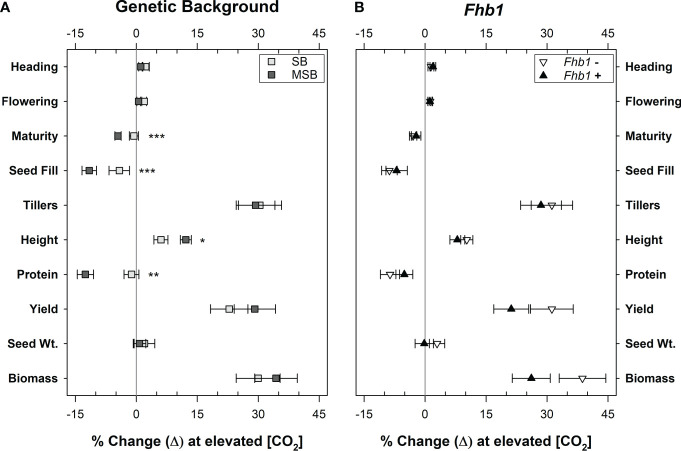
Percent change in wheat characteristics due to growth at elevated CO_2_, by genetic background or the presence of the *Fhb1* QTL. Groups are **(A)**, wheat from either a moderately susceptible (MSB) or susceptible (SB) genetic background (*n* = 24) or **(B)**, wheat with, or without, the *Fhb1* QTL (*n* = 24). Points represent the mean percent change by group. Error bars represent standard error. Change in plant developmental timings, as defined by the number of days till plants achieved Heading, Flowering, Maturity and the total days of Seed Fill. Change in productivity measures, as defined by the numbers of wheat Tillers, plant Height, grain Protein, Yield per plant, the average seed weight (Seed Wt.), above ground biomass per plant (Biomass). Asterisks (*, *, ***) denote statistically significant differences in plant characteristics at elevated CO_2_ (P<0.05, P<0.01, P<0.0001, respectively), as determined by a Student’s t Test (*n* = 24).

Neither the presence of the *Fhb1* QTL in SB, nor the absence of the *Fhb1* QTL in MSB wheat had any significant effect on plant growth, development, or yield characteristics in ambient or elevated CO_2_ (**Figure 5B**). Above ground biomass accumulation at elevated CO_2_ appeared to be impacted by *Fhb1*, but the effect was not statistically significant at an alpha level of 0.05 (P = 0.093). Wheat genetic background was the significant contributing factor in determining plant response to elevated CO_2_, as protein loss was worsened by growth at elevated CO_2_ in MSB, compared with SB (**Figure 1**, **5A**).

Furthermore, a principal component analysis of the wheat traits showed that the near isogenic lines were closely clustered, regardless of the presence of the *Fhb1* QTL (**Supplementary Figure 2**). When determining which controlled variables, i.e. CO_2_, genetic background, or *Fhb1*, were most responsible for the variance in the analysis, both genetic background (P<0.0001) and CO_2_ (P<0.0001) were found to be significant and accounted for 20% and 15% of the variance, respectively. However, the *Fhb1* QTL was not significant (P = 0.611), and only accounted for 0.4% of the variance in the analysis. Therefore, the presence of *Fhb1* had no significant impact on wheat growth and productivity.

## Discussion

Our results demonstrate that the *Fhb1* QTL was not associated with grain protein content loss in wheat grown at elevated CO_2_. However, we found that wheat from the Sumai 3/Stoa RIL 63–4//MN97448 pedigree suffered severe grain protein loss at elevated CO_2_. The Sumai 3 cultivar and its derivatives have been extensively utilized as a source of FHB resistance; however the cultivar has poor agronomic traits and breeders often have difficulty obtaining derivative breeding lines with acceptable performance (Bai et al., 2018; Zhu et al., 2019; Zhang et al., 2021). Resistance traits often incur a fitness cost, as resources used for plant self-protection become unavailable for growth or reproduction (Brown and Rant, 2013). Identifying which genes, or polygenes, are responsible for a trait is costly, difficult, and time consuming; evermore so when determining how disease resistance tradeoffs are balanced with crop performance. This defense trade-off paradigm, particularly with FHB resistance, often means the introgression of traits that only provide moderate disease resistance but are frequently associated with reduced crop performance, diminished grain protein and grain quality (McCartney et al., 2007).

However, we found that *Fhb1* had no negative impact on the agronomic traits assessed in this study; this is consistent with previous reports on wheat that had incorporated *Fhb1* from a number of Chinese donor cultivars highly resistant to FHB (Li et al., 2019; Zhang et al., 2021). It should be noted, *Fhb1* has been observed to negatively impact grain protein content in wheat, particularly when coupled with the *Fhb5* QTL (Brar et al., 2019a; Brar et al., 2019b). Sumai 3, the key parental line providing resistance factors for the genotypes in this study (**Table 1**), contains *Fhb1*, *Fhb2*, *Fhb5* and other minor alleles associated with cell wall thickening and Type II FHB resistance (Brar et al., 2019a). The *Fhb5* resistance loci is associated with Type I resistance, or the prevention of the initial fungal infection (Xue et al., 2011); the *Fhb1 and Fhb2* QTLs provides Type II resistance which improves wheat resistance to pathogen spread (Bai et al., 1999; Buerstmayr et al., 2002; Cuthbert et al., 2006; Yang et al., 2006). The *Fhb5* QTL has been associated with significant reductions in grain protein content when introgressed into wheat cultivars (McCartney et al., 2007; Brar et al., 2019b). However, the *Fhb2* QTL may have also been partially responsible for alterations in grain protein content, but it is currently unclear due to differences in trait conditions and wheat genetic backgrounds (Zhang et al., 2021). Further research is required to determine whether, or which, FHB resistance factor is responsible for the grain protein loss observed in MSB wheat at elevated CO_2_.

Preventing grain protein loss is particularly important since the utility of wheat flour is predominately determined by grain protein content, as flour hydration forms a viscoelastic dough where gluten protein structure sets and determines the final processing characteristics and texture; high protein flours are chiefly utilized for breads and pastas, while lower protein flours are typically used for cakes, cookies, and pastries (Delcour et al., 2012). The large decreases in grain protein content observed at elevated CO_2_ represents a concerning threat to future food quality and nutritional integrity. Environmental factors such as CO_2_ concentration, as well as abiotic and biotic stresses, particularly during the critical spike formation and seed development phases, can impact yield and grain protein content to varying degrees (Fernando et al., 2014; Wang and Liu, 2021). Abiotic stresses, such as heat and drought, will reduce yield due to failures in photosynthetic competency and a lack of photosynthate during seed fill, resulting in reduced seed size, mass, total grain carbohydrate and overall yield (Begcy and Walia, 2015). Grain protein content is proportionally increased due to the inability to remobilize soluble carbohydrates, but the functional protein quality and total harvestable protein is overwhelming reduced in severe heat and drought stress (Saint Pierre et al., 2008; Farooq et al., 2011).

In contrast, rising atmospheric CO_2_ can dramatically alter the primary metabolism of C3 photosynthetic crops, with increased photosynthetic rates and grain carbohydrate deposition (Broberg et al., 2017). As observed in this study, grain protein content loss was not due to a failure of seed development or stunted seed size, as the average seed weight was not affected by elevated CO_2_. The stable seed weight, even at elevated CO_2_, is consistent with the wheat being sink-limited during seed fill, rather than source-limited, *i.e.*, the available photosynthate and remobilized nutrients exceeded the sink demand of the forming seed (Borrás et al., 2004). Our results demonstrated that overall yields significantly increased in all genotypes at elevated CO_2_ (**Figure 4** & **5**), a consistent response of C3 photosynthetic crops (Ainsworth and Long, 2021). The additional photosynthate from enhanced photosynthetic carbon assimilation at elevated CO_2_ is typically utilized for greater vegetative growth, and then devoted to additional seed carrying capacity (Hay et al., 2017). In wheat, the plants produce additional tillers at elevated CO_2_ (Hay et al., 2022), consistent with the results of the current study. The most important component of a healthy crops’ yield is the total number of seeds in the cultivated area (Borrás et al., 2004). Even though modern wheat cultivars reflect exceptional breeding progress in yield improvements they are still considered more sink than source limited, due in part to inadequate seed number and size (Foulkes et al., 2011).

Furthermore, the decline of grain protein content (**Figure 1**) was not due to a lack of nitrogen uptake or availability, as the total harvestable grain protein per plant (g protein/plant) was greater due to increased yields at elevated CO_2,_ consistent with previous studies (Ziska et al., 2004). Rather, grain protein was likely being overwhelmed by the amount of carbohydrate deposited in the grain during seed fill, as observed in our previous report (Hay et al., 2022). Seed nitrogen is predominately (65%) assimilated pre-anthesis and is remobilized from vegetative tissue, starting just before or immediately after anthesis, as photosynthetic machinery, chloroplasts, and other cellular structures are disassembled for transport (Zhou et al., 2016). Wheat with low harvest index and poor sink strength had down regulated amino acid assimilation and a depletion in N, NO_3-_, and amino acid content, but up-regulated starch synthesis; this resulted in the downregulation of photosynthesis and reduced plant growth response to elevated CO_2_ (Aranjuelo et al., 2013). However, a lack of proper nitrogen remobilization coupled with the impairment of nitrate uptake and assimilation did not impact yield increases at elevated CO_2_, but instead directly affected grain protein accumulation (Pleijel and Uddling, 2012).

Furthermore, as photosynthate builds up in the leaf tissue, due to insufficient seed sink capacity, the accumulation of leaf sugars promotes the onset of senescence (Wingler et al., 1998). The early termination of grain filling can start due to a loss of sink activity, rather than a lack of assimilate during seed fill (Kim et al., 2011). In our study, we found that the MSB wheat had significantly reduced time for seed fill at elevated CO_2._ It is not clear that this was the cause of reduced nitrogen mobilization, but it is clearly correlated (**Figure 3**). In the plant species *Ricinus communis*, phloem carbon export from leaves was significantly greater at night in elevated CO_2,_ but plants remained sink limited during the day, regardless of atmospheric CO_2_ (Grimmer and Komor, 1999). The phloem vasculature connects source and sink tissues but it is tightly regulated and very sensitive to environmental conditions, which can drastically change carbon allocation to sinks (Lemoine et al., 2013). The loss of grain protein content in MSB may have been caused by a more vigorous CO_2_ response which altered carbon export in relation to nitrogen remobilization from source to sink tissues. Additionally, there was no significant reduction in yield or average seed weight compared with SB, suggesting that the MSB wheat had exhausted their seed sink capacity, causing the early onset of maturity. Efforts to simply increase the seed sink size may negatively impact protein quality, and therefore it is essential to investigate the nitrogen partitioning dynamics during seed fill (Bertheloot et al., 2008). Additional research is underway to determine how genes associated with carbon/nitrogen metabolism and transport are differentially impacted by elevated CO_2_ in varying wheat genetic backgrounds.

Though MSB wheat genotypes did experience a significant reduction in grain protein content at elevated CO_2_, we can conclude that this was not due to the presence of the *Fhb1* QTL. *Fhb1* did not negatively impact wheat development, growth, productivity, nutritional integrity nor did it alter plant response to elevated CO_2_. This research should provide plant breeders confidence in the continued utilization of *Fhb1* for enhancing FHB resistance in wheat. However, it is concerning that some wheat genetic backgrounds will suffer more severe nutrient and quality losses with rising CO_2._ While our current study was focused on evaluating grain protein content, we are actively investigating how elevated CO_2_ impacts gluten composition and protein functionality in additional wheat cultivars. Identifying climate resilient and disease resistant wheat traits is essential for securing future food security.

## Data availability statement

The raw data supporting the conclusions of this article will be made available by the authors, without undue reservation.

## Author contributions

WH planned, designed, and conducted the research efforts. Additionally, he was the primary manuscript author. JA and DG assisted in the experimental design and contributed to writing and editing the manuscript. SM assisted in sample analysis and manuscript editing. MV supervised the research, assisted with plant evaluations, and contributed to writing and editing the manuscript. All authors contributed to the article and approved the submitted version.

## Funding

This work was funded by the United States Department of Agriculture.

## Acknowledgments

We would like to thank Nathan Kemp and Jennifer Teresi for technical assistance with plant care and grain harvest.

## Conflict of interest

The authors declare that the research was conducted in the absence of any commercial or financial relationships that could be construed as a potential conflict of interest.

## Publisher’s note

All claims expressed in this article are solely those of the authors and do not necessarily represent those of their affiliated organizations, or those of the publisher, the editors and the reviewers. Any product that may be evaluated in this article, or claim that may be made by its manufacturer, is not guaranteed or endorsed by the publisher.

## Disclaimer

This work was funded by the United States Department of Agriculture. Mention of trade names or commercial products in this publication is solely for the purpose of providing specific information and does not imply recommendation or endorsement by the U.S. Department of Agriculture. Authors have no conflicts of interest to declare. USDA is an equal opportunity provider and employer.

## Abbreviations

FHB, Fusarium head blight; a[CO_2_], Ambient CO_2_; e[CO_2_], Elevated CO_2_; SB, Susceptible genetic background; MSB, Moderately susceptible genetic background; QTL, Quantitative trait loci.
