# Supplementary material for: Fhb1 disease resistance QTL does not exacerbate wheat grain protein loss at elevated CO2
Source: Front Plant Sci. 2022 Nov 28;13:1034406. doi: 10.3389/fpls.2022.1034406 (PMC9742602; doi:10.3389/fpls.2022.1034406)
Supplement: Supplementary file 1 [file DataSheet_1.docx]

Supplementary Material

***Fhb1* disease resistance QTL does not exacerbate wheat grain protein loss at elevated CO_2_**

**William T. Hay^1*^, James A. Anderson^2^, David F. Garvin^2^, Susan P. McCormick^1^, Martha M. Vaughan^1^**

^1^USDA, Agricultural Research Service, National Center for Agricultural Utilization Research, Mycotoxin Prevention and Applied Microbiology Unit, 1815 N, University Street, Peoria, IL 61604, USA.

^2^Department of Agronomy & Plant Genetics, University of Minnesota, St. Paul, MN 55108, USA

*** Correspondence:**Corresponding Author
[William.Hay@usda.gov](mailto:William.Hay@usda.gov), 309-681-6361; ORCID ID: 0000-0001-8784-6591

Disclaimer: This work was funded by the United States Department of Agriculture. Mention of trade names or commercial products in this publication is solely for the purpose of providing specific information and does not imply recommendation or endorsement by the U.S. Department of Agriculture. Authors have no conflicts of interest to declare. USDA is an equal opportunity provider and employer.

## Supplementary Figures


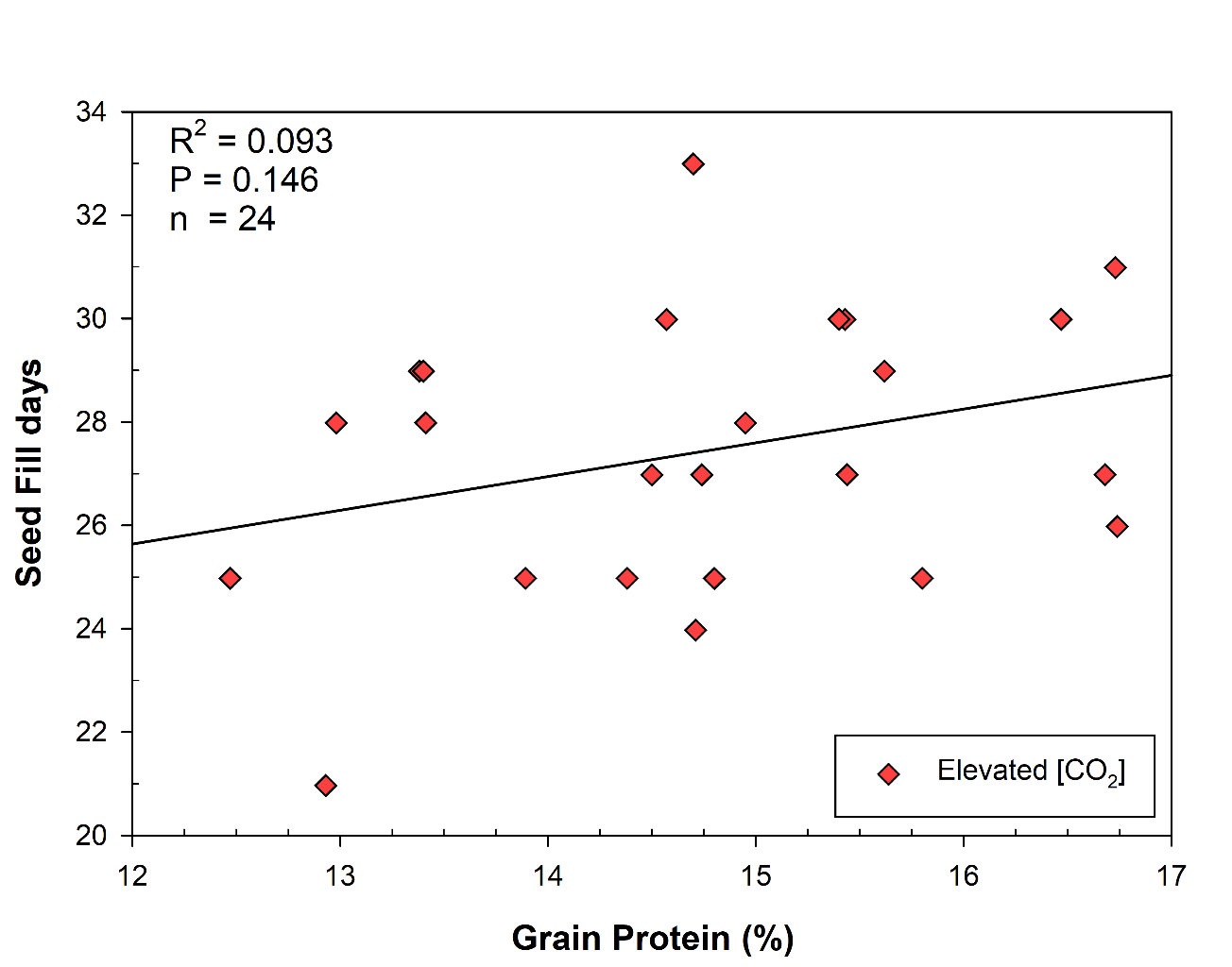


**Supplementary Figure 1**. Linear correlations between seed fill days and grain protein only in wheat at elevated CO_2,_ in moderately susceptible wheat cultivars. Linear fits were produced, and the analysis of variance was performed using JMP V15.0.


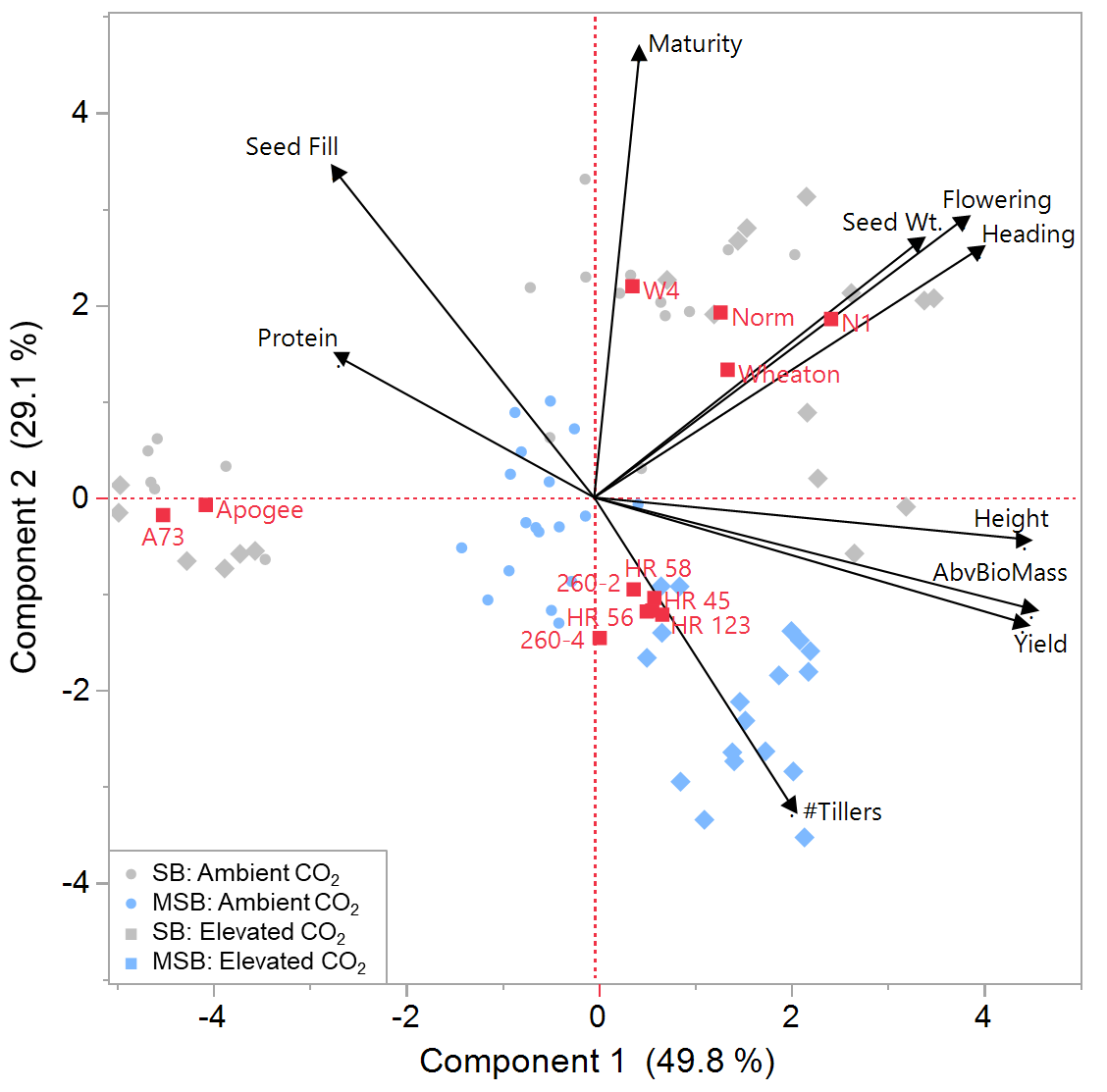


**Supplementary Figure 2**. Principal component analysis (PCA) of wheat cultivars grown at ambient and elevated CO_2_. The biplot displays each cultivar in relation to the two principal components which account for the greatest variance of wheat agronomic data. Coordinates for each cultivar observation are calculated in an orthoganal linear transformation by the pair of principal components. The centroid position of each cultivar is represented by a red square with a corresponding cultivar label; cultivar details can be found in Table 1. Arrows represent the influence of each independent variable on the principal components.
